# The Influence of DNA Extraction and Lipid Removal on Human Milk Bacterial Profiles

**DOI:** 10.3390/mps3020039

**Published:** 2020-05-15

**Authors:** Anna Ojo-Okunola, Shantelle Claassen-Weitz, Kilaza S. Mwaikono, Sugnet Gardner-Lubbe, Heather J. Zar, Mark P. Nicol, Elloise du Toit

**Affiliations:** 1 Division of Medical Microbiology, Department of Pathology, Faculty of Health Sciences, Observatory 7925, University of Cape Town, Cape Town 7700, South Africa; tellafiela@gmail.com (S.C.-W.); mark.nicol@uwa.edu.au (M.P.N.); elloisedutoit@gmail.com (E.d.T.); 2 Computational Biology Group and H3ABioNet, Department of Integrative Biomedical Sciences, Observatory 7925, University of Cape Town, Cape Town 7700, South Africa; kilazasmsn24@gmail.com; 3 Department of Science and Laboratory Technology, Dar es Salaam Institute of Technology, 11000 Dar es Salaam, Tanzania; 4 Department of Statistics and Actuarial Science, Faculty of Economic and Management Sciences, Stellenbosch University, Matieland 7602, Stellenbosch, South Africa; slubbe@sun.ac.za; 5 Institute of Infectious Disease and Molecular Medicine, Faculty of Health Sciences, Observatory 7925, University of Cape Town, Cape Town 7700, South Africa; heather.zar@uct.ac.za; 6 SAMRC Unit on Child & Adolescent Health, Observatory 7925, University of Cape Town, Cape Town 7700, South Africa; 7 Department of Pediatrics and Child Health, Red Cross War Memorial Children’s Hospital, Rondebosch, Cape Town 7700, South Africa; 8 School of Biomedical Sciences, Division of Infection and Immunity, The University of Western Australia, M504, Perth, WA 6009, Australia

**Keywords:** 16S rRNA gene sequencing, bacterial profiles, DNA extraction, human milk, skim milk, whole milk

## Abstract

Culture-independent molecular techniques have advanced the characterization of environmental and human samples including the human milk (HM) bacteriome. However, extraction of high-quality genomic DNA that is representative of the bacterial population in samples is crucial. Lipids removal from HM prior to DNA extraction is common practice, but this may influence the bacterial population detected. The objective of this study was to compare four commercial DNA extraction kits and lipid removal in relation to HM bacterial profiles. Four commercial DNA extraction kits, QIAamp^®^ DNA Microbiome Kit, ZR Fungal/Bacterial DNA MiniPrep™, QIAsymphony DSP DNA Kit and ZymoBIOMICS™ DNA Miniprep Kit, were assessed using milk collected from ten healthy lactating women. The kits were evaluated based on their ability to extract high quantities of pure DNA from HM and how well they extracted DNA from bacterial communities present in a commercial mock microbial community standard spiked into HM. Finally, the kits were evaluated by assessing their extraction repeatability. Bacterial profiles were assessed using Illumina MiSeq sequencing targeting the V4 region of the 16S rRNA gene. The ZR Fungal/Bacterial DNA MiniPrep™ and ZymoBIOMICS™ DNA Miniprep (Zymo Research Corp., Irvine, CA, USA) kits extracted the highest DNA yields with the best purity. DNA extracted using ZR Fungal/Bacterial DNA MiniPrep™ best represented the bacteria in the mock community spiked into HM. In un-spiked HM samples, DNA extracted using the QIAsymphony DSP DNA kit showed statistically significant differences in taxa prevalence from DNA extracted using ZR Fungal/Bacterial DNA MiniPrep™ and ZymoBIOMICS™ DNA Miniprep kits. The only difference between skim and whole milk is observed in bacterial profiles with differing relative abundances of *Enhydrobacter* and *Acinetobacter*. DNA extraction, but not lipids removal, substantially influences bacterial profiles detected in HM samples, emphasizing the need for careful selection of a DNA extraction kit to improve DNA recovery from a range of bacterial taxa.

## 1. Introduction

There is a growing interest in the role that human milk (HM) microbes play in infant and maternal health. The HM microbiota has been shown to have a role in the development of the infant gut bacteriome and in promoting programming of the immune system [1,2]. Randomized clinical trials have shown that the clinical signs of mastitis were alleviated among women with staphylococcal lactational mastitis after the oral administration of HM-derived *Lactobacillus salivarius* and *Lactobacillus gasseri* strains as compared with the control group who ingested the placebo; HM bacteria may therefore serve as an alternative treatment for lactational infectious mastitis caused by *Staphylococcus aureus* [3,4]. 

Studies characterizing the bacterial diversity in HM initially were based on culture-dependent techniques, which had several limitations including detection of only viable organisms and being labor-intensive [5,6,7]. Culture-independent molecular techniques, enabled by next-generation sequencing (NGS), can profile bacteria in complex environments and provide detailed phylogenetic information [8,9]. For this, however, high-quality genomic DNA that is representative of the microbial communities is required. 

The optimization of DNA extraction methods has been documented for samples such as feces, vagina, soil, bovine milk, saliva and colonic tissue [10,11,12,13,14,15,16,17], with only a recent study having been conducted in HM samples [18]. This is crucial as methodological variation such as the use of different DNA extraction kits may impact microbial community profiling [19]. Moreover, the effect of removing the lipids layer of milk prior to DNA extraction is unknown, even though this approach is commonly used [20,21,22]. Lipids-rich tissue can cause difficulties in DNA extraction due to lipids interfering with tissue disruption or by influencing the chemistry of the DNA isolation buffers [23]. 

Optimization of DNA extraction from HM is necessary as HM is known to have a relatively low bacterial biomass, and interfering substances (such as proteins) pose a challenge to the extraction of large amounts of quality DNA [17]. In addition, the HM bacteriome has been reported to contain a variety of Gram-positive and Gram-negative bacterial species [20] with differing cell wall composition, which makes some species more difficult to lyse than others. Improper lysis of these two groups of bacteria may result in a biased representation of the bacterial community present in HM samples. Hence, methods need to be tested for their effectiveness, efficiency to lyse bacterial cells, and the quality of the extracted DNA. 

The aim of this study was to compare and evaluate the extraction of bacterial genomic DNA from HM samples using four commercial DNA extraction kits. Selection of kits used in this study was based on their availability and prior use for HM bacteriome studies. We also incorporated a mock microbial community with predetermined DNA ratios from a mixture of bacterial species to assess bias of the DNA extraction kits. Furthermore, whole milk (WM) and skim milk (SM) were compared to determine whether the removal of the lipids layer affected the bacterial population detected in HM.

## 2. Material and Methods

### 2.1. Subjects and Sample Collection

HM samples were collected from ten healthy lactating women residing in Cape Town, South Africa after their consent was obtained. The women were asked to wash their hands, their nipples and surrounding area with soap and water. Milk was collected manually by hand expression into a 50 mL sterile collection bottle after discarding the first few drops. After collection, the samples were transported on ice, aliquoted, and stored at −20 °C until further processing. This study received ethical approval from the University of Cape Town Human Research Ethics Committee, South Africa (HREC REF: 649/2016).

### 2.2. Methods of DNA Extraction

**Figure 1:**
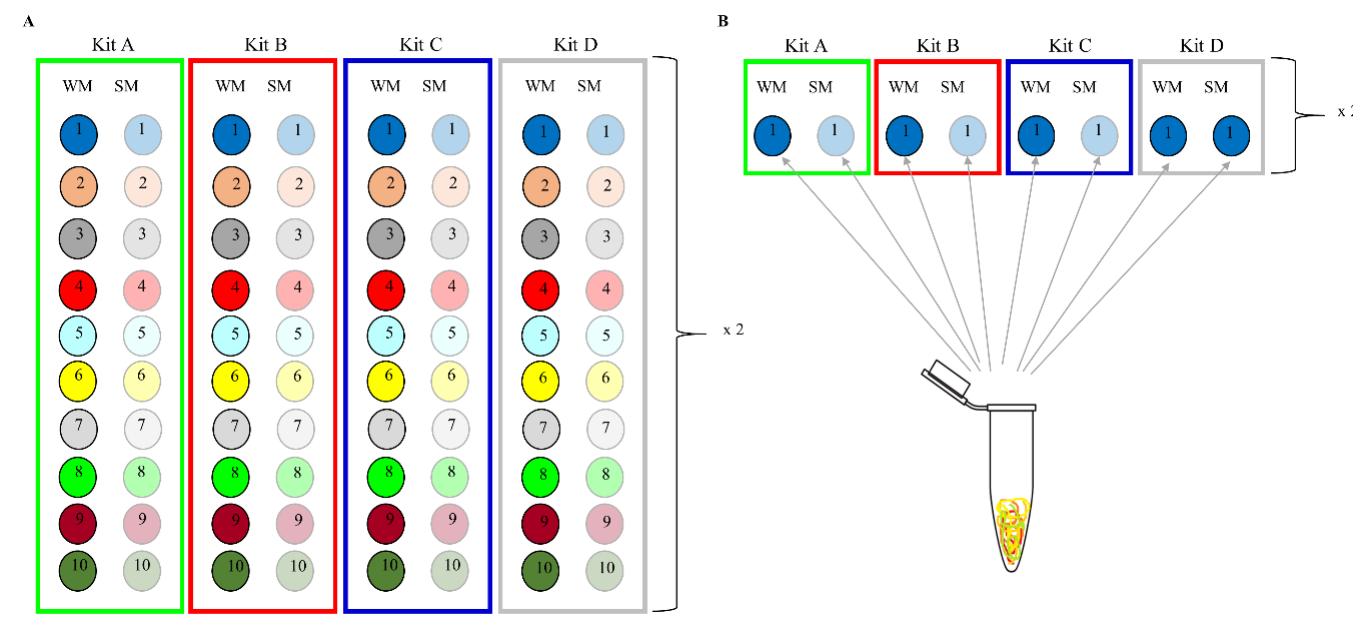
Human milk samples collection per milk type and DNA extraction method. (**A**) DNA extraction from un-spiked skim milk and whole milk samples. (**B**) DNA extraction from spiked skim milk and whole milk samples.

Each of the ten HM samples was processed as un-spiked SM (n = 10) and WM (n = 10). DNA was extracted in duplicate from each SM and WM sample, using 4 different kits (total number of extracts = 160, Figure 1A). In addition, as an extraction control, one HM sample was divided into SM and WM, with each 1 mL sample spiked with 75 µL (1 × 10^9^ cells) of Zymobiomics Microbial Community Standard (ZMCS), (Catalogue no. D6300, Zymo Research Corp., Irvine, CA, USA). As per manufacturer’s specifications, the genomic DNA abundance (%) for each bacteria species is 12% while the microbial operational taxonomic unit (OTUs) relative abundances are *Pseudomonas* spp. (4.6%), Enterobacteriaceae 1 (11.3%), Enterobacteriaceae 2 (10.0%), *Listeria* spp. (15.9%), *Staphylococcus* spp. (13.3%), *Lactobacillus* spp. (18.8%), *Enterococcus* spp. (10.4%) and *Bacillus* spp. (15.7%) (Table S1). Commercial ZMCS was employed as the mock community because it is readily available and affordable and also manufactured by Zymo Research Corp., Irvine, CA, USA, the same company which manufactured two of the DNA kits assessed in the study. DNA was extracted from replicates (on consecutive days) of the same spiked sample with each of the 4 kits (n = 16) (Figure 1B). 

HM samples were homogenized by vortexing and SM was prepared by adapting a previously published protocol [24]. In brief, the samples were centrifuged at 3500× *g* for 20 min at −10 °C, and the fat layer removed by a 10 μL disposable inoculation loop. The supernatant was thereafter centrifuged in the same tube at 7600× *g* for 10 min at room temperature, and the cell pellet used for further processing. The pellet from WM was prepared by centrifugation of the original milk sample at 7600× *g* for 10 min at room temperature. DNA was extracted from un-spiked HM samples (n = 160) and spiked HM samples (n = 16) using the recommended starting volume of the four different commercial DNA kits described below:

Kit A (QIAamp^®^ DNA Microbiome Kit), (Qiagen, Hilden, Germany): 500 μL Buffer AHL (host cell lysis buffer) was added to either 1 mL of WM (or SM) for DNA extraction from WM and SM respectively. The pellet obtained after centrifugation was used for further processing.

Kit B (ZR Fungal/Bacterial DNA MiniPrep™), (Zymo Research Corp., Irvine, CA, USA): The pellet obtained from centrifugation of 1mL of WM (or SM), was resuspended in 250 μL of the resultant supernatant before proceeding to add 750 μL Lysis Solution (Zymo Research Corp., Irvine, CA, USA) to the tube.

Kit C (QIAsymphony DSP DNA), (Kit Qiagen, Hilden, Germany): The pellet obtained from centrifugation of 1 mL of WM (or SM) was resuspended in 250 μL of the resultant supernatant. An “off-board” mechanical lysis step followed as recommended by the manufacturer and as previously described [11], using 750 μL Lysis Solution and ZR BashingBead^TM^ (Zymo Research Corp., Irvine, CA, USA). Following mechanical lysis, the lysate was centrifuged at 5800× *g* for 1 min, and 400 µL of the supernatant was used for DNA extraction on the QIAsymphony^®^ SP instrument (Qiagen, Hombrechtikon, Switzerland). 

Kit D (ZymoBIOMICS™ DNA Miniprep Kit), (Zymo Research Corp., Irvine, CA, USA): the pellet obtained from centrifugation of 1 mL of WM (or SM), was resuspended in 250 μL of the resultant supernatant before proceeding to add 750 μL ZymoBIOMICS™ Lysis Solution (Zymo Research Corp., Irvine, CA, USA) to the tube.

An elution volume of 50 µL was used for all the kits except kit C, in which the minimum elution volume was 60 µL as set by the supplier. For homogeneity and to ensure higher concentrations of DNA samples (as recommended by the manufacturer), 50 µL elution volume was used for Kit D. With the exception of Kit D, which was eluted in DNase/RNase Free Water, DNA eluted from other kits were extracted in elution buffers. All bead-beating steps were performed in the TissueLyser LT™ (Qiagen, FRITSCH GmbH, Idar-Oberstein, Germany) at a frequency of 50 Hz for 5 min.

### 2.3. DNA Quantification

The concentration and purity of DNA were measured using a NanoDrop™ ND-2000c Spectrophotometer (Thermo Fisher Scientific Inc., MA, USA). DNA yield was obtained by multiplying the DNA concentration by the final elution volume. The DNA yield from all samples was also assessed by 16S quantitative polymerase chain reaction (qPCR) using a protocol previously described [25]. Each 30 µL PCR reaction contained 2.5 µL DNA template, 1 µL of 0.166 µM probe, 15 µL of SensiFAST™ Probe No-ROX (Catalogue no. BIO-86020, Bioline, MA, USA), 9.5 µL of MilliQ water and 1 µL each of 0.333 µM forward and reverse primer with conditions as described (Table S2A). The qPCR was carried out on a 7500 Fast Real-Time PCR System (Applied Biosystems, Foster City, CA, USA). DNA was stored at −20 °C until further processing.

### 2.4. Extraction and Sequencing Controls

The ZMCS was extracted using all four kits and served as a positive extraction control. DNA extraction on all HM samples was done in duplicate on two consecutive days using the respective kits to evaluate extraction repeatability. In addition, DNA extracts from two samples were randomly selected for repeat processing (library preparation and sequencing) to evaluate repeatability of steps following DNA extraction.

In low biomass samples such as HM samples, a portion of sequence reads may result from exogenous DNA contributed by reagent contaminants used during the process of DNA extraction and 16S rRNA gene library preparation. To allow in silico correction for contamination, cyanobacteria (*Arthrospira spirulina*) DNA extract obtained from a pure culture of Cyanobacterium (*Arthrospira spirulina*) was spiked into DNA extracts from each of the respective elution buffers (negative extraction control) at a 16S rRNA gene concentration similar to that of HM samples as assessed by qPCR. Since negative controls have little or no “competing” naturally present bacterial DNA, amplification of this small amount of background DNA may lead to overestimation of the contribution of contaminants to bacterial profiles. We compensated for this effect by spiking an amount of known bacterial DNA into control samples at an equivalent concentration to that found in HM samples. These “cyanobacteria-spiked-elution buffers” were included in the library preparation and sequencing steps alongside the samples. 

### 2.5. 16S Ribosomal Ribonucleic Acid (rRNA) Amplicon Library Preparation

A two-step amplification approach described by Wu and colleagues [26] was employed to avoid PCR amplification biases associated with the use of adapter and index sequences. In the first PCR reaction, the hypervariable V4 region of the 16S rRNA gene was amplified using primers and PCR cycling conditions as previously described [27,28] (Table S2B). Each 25.25 µL PCR reaction contained 12.5 µL 2× MyTaq™ HS Mix (BIO-25046), 2 µL of 0.8 µM forward and reverse primers, 1 µL of MilliQ water, 0.75 µL dimethyl sulphoxide (catalog no D4540, Sigma-Aldrich^®^, St. Louis, MO, USA) and 7 µL DNA template. 

In the second PCR reaction, the same reagents were used as above, except that the template was 7 µL of the amplicon product from the first PCR reaction, and the reverse primers contained Illumina adapters and various unique index sequences at the 3’ end for each sample [29]. The PCR conditions are the same with the short PCR run as described except that 30 cycles were conducted in the 2nd PCR run [27] (Table S2C). 

Amplicon products were cleaned with Agencourt SPRIPlate 96 super Magnet Plate, and QuantiFluor™ dsDNA System was used to quantify cleaned amplicons [27,28]. The integrity of the cleaned amplicons was checked by gel electrophoresis. Briefly, 5 µL of each cleaned amplicon was analyzed on a 2% agarose gel containing 1% ethidium bromide. Amplicons were normalized by pooling at an equimolar concentration of 100 ng and purified using Agencourt AMPure system (Beckman Coulter, UK). Pooled library was extracted on 1.5% agarose gel. QIAquick Gel Extraction Kit (Qiagen, MA, USA) was used for gel purification with the following minor modification to manufacturer’s protocol. The elution buffer, Tris-EDTA buffer (pH 8.0), was heated at 70 °C to improve amplicon recovery (step 13). Qubit^®^ dsDNA BR Assay Kit was used for final quantification of the pooled 16S library.

### 2.6. 16S Ribosomal Ribonucleic Acid (rRNA) Gene Sequencing

The pooled 16S library was paired-end sequenced on the Illumina^®^ MiSeq™ platform using the MiSeq Reagent v3 kit, 600 cycles (Illumina, CA, USA). The quality control steps entailed (1) the quantitation of adapter-ligated dsDNA using the KAPA Library Quantification Kits (Illumina^®^) (KAPA Biosystems, MA, USA) and (2) analysis of fragment size of the pooled library with the Agilent High-Sensitivity (HS) DNA Kit (Agilent Technologies, Santa Clara, CA, USA). The library pool was thereafter diluted to 4 nM using Buffer EB (Qiagen, Hilden, Germany), and denatured and neutralized using 0.2 N NaOH and HT1 Buffer (Illumina^®^). A final library dilution was prepared at 5.5 pM, which was loaded to the sequencer according to the manufacturer’s instructions [30], alongside the sequencing control (PhiX library) spiked into the 16S library at 15% (v/v).

### 2.7. Bioinformatics Workflow

The sequencing quality of FASTQ files was assessed using FASTQC (v0.10.1) package [31]. Forward and reverse sequences were then merged using UPARSE (v7.0.1090), allowing 3 mismatches in overlaps (fastq_maxdiff set to 3), followed by quality filtering using USEARCH9 fastq_filter (sequences truncated to 250bp). Reads with a maximum expected number of error >0.1 were discarded (fastq_maxee set to 0.1) [32]. De-replication and selection of sequences occurring more than once was performed by sortbysize command in USEARCH9. Clustering of sequences into operational taxonomic units (OTUs) (with a clustering radius of 3) was done using USEARCH9 cluster_otus command. The USEARCH9_uchime2_ref tool was used to detect and remove chimeras, and OTU counts were obtained using USEARCH9 usearch-global [33]. 

Decontamination of HM samples was based on type of kit used and was done by first removing cyanobacteria sequences from the four “cyanobacteria-spiked-NTC” controls. Sequences remaining after the removal of cyanobacteria sequences were identified as “contaminant sequences”. The latter were screened against HM samples by aligning HM sample sequences to spiked control sequences at 100% similarity using align_seq.py, based on PyNAST [34]. An average number of reads was calculated for each of the “contaminant sequences” matching at 100% similarity to HM sample sequences. “Contaminant sequences” were removed from HM samples by removing the average number of reads calculated from the four “cyanobacteria-spiked-NTC” controls.

Further processing of data was performed using Quantitative Insights Into Microbial Ecology (QIIME) 1.9.1 suite of software tools [35]. OTU picking occurred at 97% sequence similarity, and taxonomic assignment was carried out against SILVA database (Version 132.) [36] using Ribosomal Database Project (RDP) classifier (v2.2) in QIIME (v1.9.1) [35]. Rarefaction plot of Shannon diversity against sequencing depth was also generated in QIIME using alpha_rarefaction.py [35]. 

The raw sequencing reads were deposited in the NCBI Sequence Read Archive (SRA) database with accession number PRJNA510564.

### 2.8. Data Analysis

Data analysis and graphical illustrations of the data (bar plots, boxplots, dendograms) were generated in R statistical package (version 3.4.1) and R studio 1.1.456 [37]. Agglomerative cluster dendograms were generated by complete linkage hierarchical clustering [38] using the [hclust] function [39]. This hierarchical clustering method is based on the Bray–Curtis dissimilarity index [40] of the R *vegan* package [41]. Cluster dendogram was performed for all OTUs with relative abundance of >0.5%. Alpha diversity within each sample was measured using the Shannon–Weaver index with function [diversity] in the R package *vegan* [42], which measures both the richness and evenness of organisms within a given sample. Analysis of Variance (Type II tests) [43] was used to test the significant difference in alpha diversity between groups and to generate a *p*-value with a significance threshold of *p* < 0.05, while error estimates were based on Pearson residuals. Log-ratio biplots using a Bayesian prior technique for adjustments of zero counts were made as previously described [44] and employed lambda-scaling to ensure evenness in the “total spread” of the data sets [45]. Log-ratio biplots were used to show multivariate clustering patterns as they are specific for proportions/percentages [46]. 

Generalized linear models (GLM) were used to test the effect of SM and WM, and the four DNA extraction kits, on HM bacterial profiles at different taxonomy levels. The negative binomial distribution [47] in the package stats with the Quasi-Poisson family function [48] was applied to model over-dispersion. Benjamini–Hochberg method for multiple correction was used to correct all *p*-values, set at a 5% significance level, by the false discovery rate (FDR) [49]. Tukey’s Honest Significant Differences (HSD) method was used to generate a single-step multiple comparison of means procedure with 95% family-wise confidence intervals [50]. Notched box plots [51] were made to show distribution analysis of the data, as they display the 95% confidence interval for the median.

## 3. Results

### 3.1. Influence of DNA Extraction Kits and Lipid Removal on Yield and Quality of DNA Extracted from Un-Spiked Human Milk Samples

**Figure 2:**
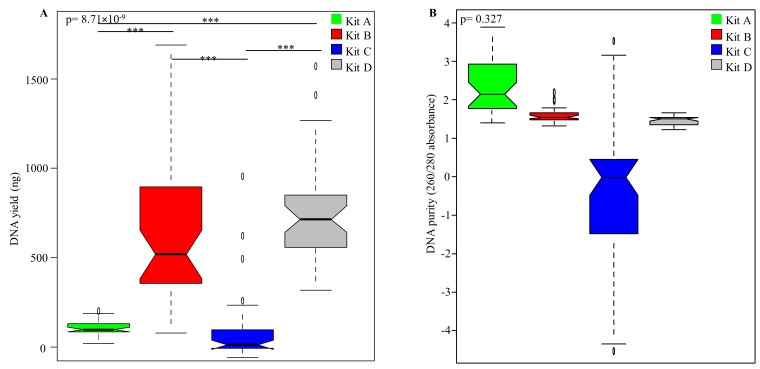
DNA yield and purity of the four different commercial kits. Notched box plots showing (**A**) the DNA yield and (**B**) the DNA purity (260/280 absorbance) obtained by using each of the four kits. The notched box signifies the 75% (upper) and 25% (lower) quartile showing the distribution of 50% of the samples. The line inside the box plot represents the median, and the notch the 95% confidence interval for the median. The whiskers (top and bottom) represent the maximum and minimum values. Outliers, which are beyond 1.5 times the interquartile range above the maximum value and below the minimum value, are shown with open circles. *** represents *p* < 0.001.

The efficiencies of four DNA extraction kits were compared based on yield and purity of the extracted DNA from un-spiked HM samples (n = 160) with NanoDrop™ ND-2000c Spectrophotometer (Thermo Fisher Scientific Inc., MA, USA) (Figure 2). A significant difference in the yield of DNA extracted was observed between the kits (*p* = 8.71 × 10^−9^) (Figure 2A). Kits B and D gave the highest DNA yield; Tukey’s HSD revealed no significant difference between these two kits (*p* = 0.96). No significant difference was observed in DNA yield when comparing SM and WM (Figure S1A). However, when comparing bacterial 16S DNA concentration from HM samples, using qPCR, no significant difference was observed between kits (*p* = 0.253) (Figure S2A). Similarly, no significant difference was observed in 16S DNA concentration when comparing SM and WM (*p* = 0.524) (Figure S2B). 

DNA purity was assessed using the 260/280 absorbance ratio measure as previously described [18], and no significant differences were observed between the four kits (*p* = 0.327) (Figure 2B), though the purity of DNA varied between the kits. Kits B and D had DNA purity closest to the recommended range of 1.8–2.0, while kit C showed a large variation in DNA purity between samples. There was no significant difference in DNA purity between SM and WM (Figure S1B).

### 3.2. Influence of DNA Extraction Kits and Lipid Removal on Bacterial Profiles Obtained from Mock Microbial Community Standard Spiked into Human Milk

To evaluate which DNA extraction kit best extracted the bacterial communities in the known ZMCS community, composition and abundance were assessed after spiking this community into WM and SM of sample 1 (extracted by each of the 4 kits in duplicate) (Figure S3B). When comparing the bacterial 16S DNA concentration in the spiked vs. un-spiked sample, >99.9% of the total 16S DNA within the spiked sample originated from the ZMCS (Table S3), and therefore, the contribution of the endogenous bacterial microbiota of this sample to the profiles generated from this sample might be negligible. The log_10_ median 16S gene copy numbers/uL are shown in Figure S4. There was a 3.8 log_10_ difference between the medians of spiked (5.7 log_10_) and un-spiked (1.9 log_10_) samples. The spiked-in DNA therefore accounted for >99.9% of the total DNA in these samples.

Hierarchical cluster analysis was used to create a dendogram of the bacterial composition of the ZMCS spiked into the HM sample alongside the community profile provided by the manufacturer (Figure S3). Bacterial profiles did not cluster based on whether DNA was extracted from WM or SM but rather based on the DNA extraction kit used. Overall, kit A showed a very different profile compared to ZMCS and the other three kits under investigation. Kit A only extracted DNA from three of the eight bacterial genera/families present in ZMCS. The three other kits (kits B, C and D) represented all the eight bacterial genera/families albeit in differing abundance. Kit C showed the widest variation in composition between replicates, with the samples clustering on different clades of the dendogram. Kit B (and some replicates for kit C) clustered closest to the ZMCS, suggesting the best representation of the microbial community standard. 

Beta diversity (Bray–Curtis dissimilarity index) was also computed to show the differences in bacterial composition between the composition of ZMCS and DNA extracted from each kit for WM and SM spiked samples. A lower beta diversity value would mean greater similarity between the composition of ZMCS and the HM sample spiked with ZMCS. For SM, kit B had the lowest beta diversity of 0.06, meaning it most closely represented the bacterial profiles of the ZMCS. For WM, the lowest beta diversity of 0.16 was seen for kits B and C (Table S4). 

**Figure 3:**
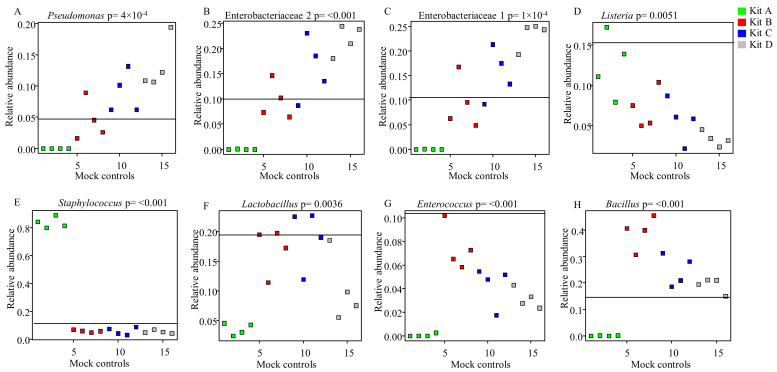
Relative abundances of bacterial taxa extracted by kits in comparison to the commercial ZMCS composition. The horizontal line in each box indicates the relative abundance of the organism in question in the commercial ZMCS as given by the manufacturer. *p* < 0.05 shows a statistically significant difference in relative abundance of each bacterial taxon between different kits. *p*-values were generated by Anova test in *stats* package of R and adjusted using Benjamini–Hochberg’s false discovery rate. ZMCS = Zymobiomics Microbial Community Standard.

**Figure 4:**
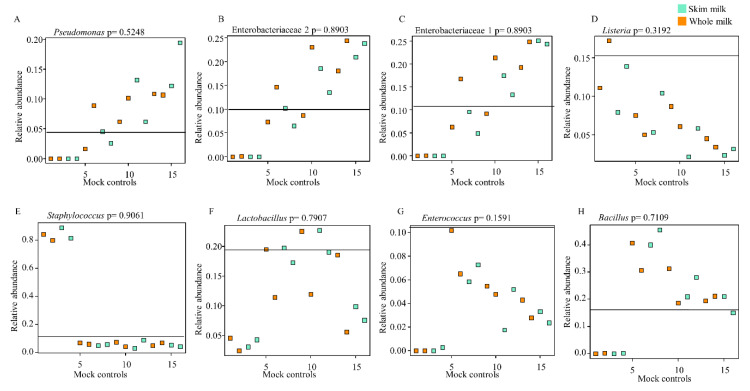
Relative abundances of bacterial taxa extracted by skim milk and whole milk in comparison to the commercial ZMCS composition. The horizontal line in each box indicates the relative abundance of the organism in question in the commercial ZMCS as given by the manufacturer. *p* > 0.05 shows a non-statistically significant difference in relative abundance of each bacterial taxon in the human milk (HM) sample spiked with ZMCS as extracted by skim milk and whole milk. *p*-values were generated by Anova test in *stats* package of R and adjusted using Benjamini–Hochberg’s false discovery rate. ZMCS = Zymobiomics Microbial Community Standard.

We further evaluated the differences in relative abundances of the eight bacterial genera/families expected from the known “theoretical” bacterial profile of ZMCS and those resulting from the extraction kits (Figure 3). Gram-negative organisms present in ZMCS were substantially under-represented in samples extracted with Kit A (Figure 3A–C). Kit B best represented the Gram-negative organisms in the known mock community with relative abundances closest to the mock community (Figure 3A–C). The relative abundances of Gram-negative organisms in the ZMCS were over-represented in DNA extracted using Kit C and Kit D (Figure 3A–C). In relation to the five Gram-positive organisms, no kit showed an ideal representation of all (Figure 3D–H). Kit B and Kit C resulted in the closest proportional representation of *Lactobacillus* spp. to the ZMCS. On the other hand, they showed a lower relative abundance of *Listeria* spp. and *Staphylococcus* spp. compared with ZMCS. There were no differences in relative abundances of taxa in ZMCS extracted from SM vs. WM (Figure 4A–H). 

**Figure 5:**
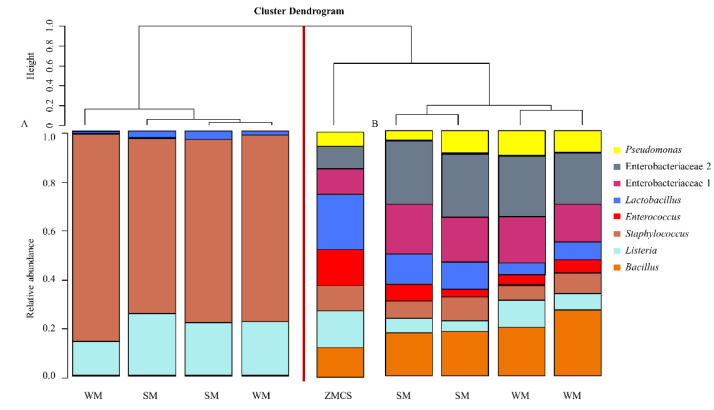
Complete linkage hierarchical clustering showing the relative abundances of bacterial taxa in the HM sample spiked with ZMCS. (**A**) Kit A DNA extraction protocol as per manufacturer’s recommendations and (**B**) Kit A DNA extraction protocol in which the initial steps (steps 1 to 4) involving degradation and digestion of host DNA were omitted. Samples were processed in duplicate. WM: whole milk, SM: skim milk, ZMCS: Zymobiomics Microbial Community Standard reference.

Due to the poor performance of kit A in extracting DNA from *Pseudomonas* spp., Enterobacteriaceae, *Enterococcus* spp. and *Bacillus* spp. in the HM sample spiked with ZMCS, the spiked samples were re-extracted with kit A; however, on this occasion, the first steps of the manufacturer’s guidelines involving the initial host DNA depletion step (benzonase treatment) were omitted—a decision based on communication with the manufacturer. The benzonase steps are intended to deplete extracellular bacterial DNA. This modification of the extraction protocol resulted in substantially improved representation of the bacteria in the ZMCS (Figure 5). However, at this point, kit A was excluded from further analysis of (un-spiked) HM samples.

### 3.3. Influence of DNA Extraction Kit and Milk Type on Bacterial Diversity in Un-Spiked HM Samples

A total of 263,475 high-quality filtered and merged reads was obtained from 160 HM samples. The number of post-filtered reads after the removal of contaminant sequences was 263,464. The median and mean sequence read count per sample were 1405 and 1670 respectively. After subsampling of 2100 reads from each sample, the alpha rarefaction curves showed that Shannon diversity plateaued at a sequence depth of 2100 (Figure S5). 

The dominant phyla in HM samples were Firmicutes and Proteobacteria, followed by a relatively low abundance of Actinobacteria and Cyanobacteria. At the genus level, the predominant bacteria were *Staphylococcus* spp., *Streptococcus* spp., *Lactobacillus* spp., *Acinetobacter* spp., and members of the family Enterobacteriaceae (Figure S6).

**Figure 6:**
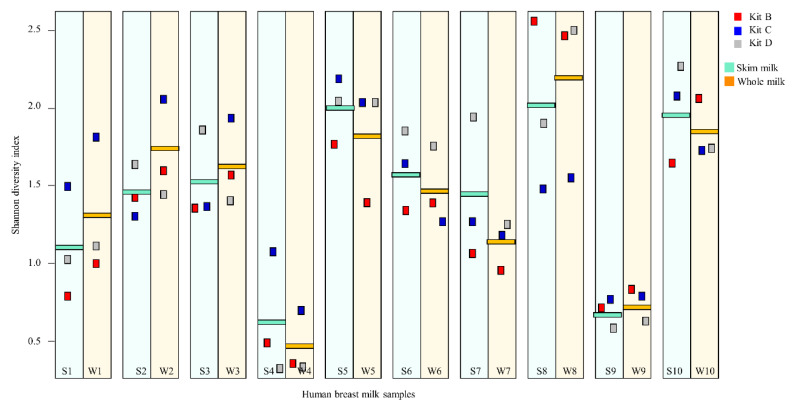
Shannon diversity index of the un-spiked HM samples based on kits and milk type. The average alpha diversity of each HM sample and its duplicate is represented by a rectangle. The horizontal cyan and orange lines represent bacterial alpha diversity from skim milk and whole milk, respectively. The alpha diversity in bacterial DNA extracted by each kit is indicated by colored boxes. Kits B, C and D are represented by red, blue and grey lines, respectively. WM = whole milk, SM = skim milk.

The influence of DNA extraction kits and extraction from SM vs. WM were compared with respect to bacterial alpha diversity (Shannon diversity index). Overall; there were no significant differences in Shannon diversity between DNA extracted by different kits (*p* = 0.11) (Figure 6). Tukey HSD multiple comparisons of means also showed no significant difference in Shannon diversity of samples between each of the kits. Similarly, there were no statistically significant differences in Shannon diversity of DNA extracted from WM vs. SM (*p* = 0.88) (Figure 6). Therefore, extraction kit and milk type did not influence the Shannon diversity of bacterial composition in HM samples.

**Figure 7:**
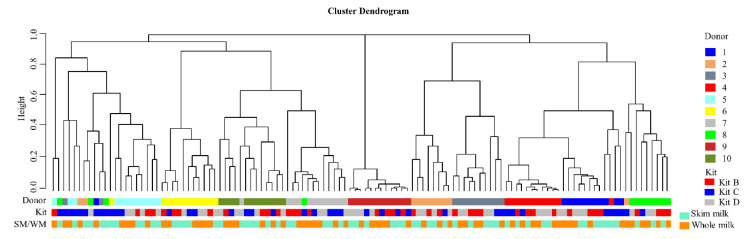
HM samples cluster primarily based on the donor. Complete linkage unsupervised hierarchical clustering based on the relative abundances of bacteria genera in un-spiked HM samples show that DNA extraction kits and milk type affects bacterial profiles less when compared to the source. W: whole milk, S: skim milk; 1–10 represents each donor; kit B: ZR Fungal/Bacterial DNA MiniPrep™, kit C: QIAsymphony DSP DNA Kit, and kit D: ZymoBIOMICS™ DNA Miniprep Kit.

Unsupervised hierarchical clustering of the relative abundances of bacteria genera in HM samples based on Bray–Curtis dissimilarity index showed clustering largely based on HM donor, rather than on the basis of DNA extraction kit used (Figure 7). 

Furthermore, exploration of bacteria with abundances > 0.5% using log ratio biplots did not show clustering of HM samples associated with either DNA extraction kit used (B, C or D) or milk type (SM or WM) either at the phylum level (Figure S7) or genus level (Figure S8A,B), suggesting that neither extraction kit nor use of WM vs. SM was a major driver of differences between samples. In contrast, clustering was observed among samples from each donor, irrespective of the kit used for extraction or whether WM or SM was extracted (Figure S7C; Figure S8C). Therefore, donor, rather than extraction kit or milk type, was the major determinant of bacterial profiles identified in HM samples.

GLM and Turkey HSD were used to compare the different taxa observed in HM samples in relation to DNA extraction kit used and between WM and SM. Table S5 summarizes the mean relative abundance of statistically significant bacteria at the different taxonomy levels for the kits and for WM and SM. Comparison of different bacterial taxa between samples processed with the different DNA extraction kits showed significant differences in relative abundances of six bacterial genera (*Ralstonia* spp., *Pseudomonas* spp., *Haemophilus* spp., *Acinetobacter* spp., OTU_52 belonging to the family Rhodobacterales, and *Lactobacillus* spp.), while WM and SM differed only for two bacterial genera, *Enhydrobacter* spp., and *Acinetobacter* spp., both belonging to the family Moraxellaceae. No significant differences in relative abundances were observed between Kit B and Kit D at any taxonomy level.

### 3.4. Repeatability of Extractions 

DNA extraction was carried out in duplicate, on consecutive days to evaluate the repeatability of each DNA extraction kit. The total read count of each OTU in each duplicate set (per kit and per milk type) was tested using multiple R-squared, representing the proportion of variance reproduced by replicating the reads. 

R^2^ ranged from 0.4751 to 0.9483 (Table S6). For WM, kit D produced the most reproducible results with R^2^ of 0.9483, followed by kit C with R^2^ of 0.7421, while kit B produced the least reproducible results with R^2^ of 0.4908. For SM, kit D produced the most reproducible results with R^2^ of 0.7976, followed by kit B with R^2^ of 0.7581, while kit C produced the least reproducible results with R^2^ of 0.4751. We observed wide variations in repeatability of SM and WM for kit B and kit C, unlike that for kit D, where repeatability was better, independently of milk type. The multiple R^2^ generated for the two sequencing controls included during the 16S library preparation was 0.9713.

## 4. Discussion

We compared different methods of bacterial nucleic acid extraction from HM prior to 16S microbiome profiling, including the effect of removing the lipids layer. To our knowledge, there are limited published studies exploring this method. The use of different DNA extraction kits on HM samples resulted in significant differences in DNA yield and purity and relative abundances of specific bacterial taxa. In addition, the DNA extraction kits represented differently the bacterial profiles in a known mock microbial community. On the other hand, lipid removal prior to DNA extraction had limited impact on bacterial profiles, only showing a small influence on the relative abundance of two specific taxa within the phylum Proteobacteria.

In agreement with previously published literature [2,20,52], HM has a vast population of bacterial communities in healthy individuals and is dominated at the phylum level by Firmicutes and Proteobacteria, followed by Actinobacteria. At the genus level, we found that *Staphylococcus* spp., *Streptococcus* spp., *Lactobacillus* spp., *Acinetobacter* spp., and members of the family Enterobacteriaceae were the dominant members. 

DNA extraction has been validated for several bacteriome studies including feces, oral cavity, turkey cecum and soil using evaluation criteria such as DNA yield and purity, DNA shearing, repeatability, quantitative PCR [5,15,53,54,55], 16S rRNA gene sequence-based taxonomic signatures [53,54,55], bacterial community fingerprints [10,19] and custom-designed phylogenetic microarray profiles [15]. However, to our knowledge, only one study has previously compared different DNA extraction methods for HM.

In this study, DNA extraction methods were evaluated based on DNA yield and purity, bacterial diversity and repeatability. HM has a high lipids content, which may present challenges in extraction of DNA for molecular testing [17,56]. Furthermore, given the low microbial biomass present in HM, it would be expected that the bacterial DNA yields would be substantially lower than those of other specimen types, for example, fecal specimens, posing a challenge for 16S rRNA-based studies [57]. Despite these potential complications, high DNA yields and within-range DNA purity were achieved by kits B and D, both from Zymo Research Corp., Irvine, CA, USA. 

Low biomass specimens are prone to contaminating microbial DNA, which is ubiquitous within DNA extraction kits, PCR reagents and other laboratory reagents [58,59]. This contaminating DNA may distort the relative abundances of microbial composition in datasets, thereby leading to erroneous interpretations [58]. We therefore mitigated this effect by in silico decontamination of our HM samples. The variation in HM bacteriome community structure experienced with spiked HM samples extracted with kit C might be due to the low DNA yield from this method, since profiles from samples with low biomass are more likely to be affected by contaminating DNA.

In addition to the above criteria, we also evaluated the kits based on representation of the bacterial profile in a commercial mock microbial community standard. Unlike environmental or human samples, which comprise an undefined number of indigenous microorganisms [5], a microbial community standard has a defined composition and abundance of known microbes. In the study by Willner and colleagues, a mock community was also used in identifying methodological contaminants, identifying detection limits and empirical cut-off values for 16S pyrosequencing in order to filter out spurious OTUs and also to compare method repeatability [60]. 

Using 16S rRNA gene sequencing, we compared the abilities of four commercial DNA extraction kits to recover DNA from bacteria from a known commercial mock microbial community containing eight bacterial species, which had been spiked into HM, by comparing the expected bacterial profiles in the HM sample spiked with ZMCS to the theoretical bacterial composition of the mock microbial community standard. Importantly, the 16S content of the spiked samples was derived primarily from the mock community. Given the 3.8 log_10_ difference in 16S copy number between spiked and un-spiked samples, one would not expect the bacterial composition of the spiked samples to be significantly affected by the indigenous bacterial community within these samples. Whilst extraction using three of the kits resulted in reasonable representation of the mock community composition, as with other studies [5,54], significant differences in bacterial profiles were shown when DNA from mock bacterial communities was extracted with different kits. 

Gram-positive organisms are more difficult to lyse; however, kits B, C and D showed an over-representation of the genus Bacillus despite an under-representation of similar hard-to-lyse bacteria like Enterococcus. In a study by Yuan et al. (2012) that evaluated six commonly used DNA extraction procedures from the Human Microbiome study, the observed relative abundance of *Lactobacillus iners* in a mock community was higher than its expected relative abundance [5]. In a previous study that evaluated the influence of four DNA extraction methods on oral bacterial profiles, only one method’s result included representation of all bacteria in the mock community [54]. A recent study that evaluated the influence of extraction methods on HM microbiota profiles observed an under-representation of *Streptococcus* spp. when compared to the expected relative abundances in the mock [18]. Overall, kits B, C and D represented all eight expected bacterial genera/families, though in differing abundance. 

Kit A performed markedly differently from the other kits. This kit had a considerably lower DNA yield, perhaps because the initial steps incorporate the depletion of human DNA to yield enriched bacterial DNA. This supports the report of Wen et al. (2016), in which a microbial DNA extraction method with pretreatment of depletion of host nucleic acid by benzonase resulted in significantly lower DNA concentrations of samples [61]. Bacterial profiles in HM spiked with the mock community and extracted with Kit A revealed a biased composition with over-representation of Gram-positive organisms, with *Staphylococcus* spp. dominating. A previous report [62], which described methods for reducing extracellular DNA in a complex respiratory sample also showed a decrease in relative abundance of Gram-negative organisms with a benzonase pretreatment. This may have been due to lysis of Gram-negative bacteria during the early extraction steps, and subsequent depletion by benzonase treatment. On repeating this extraction but omitting the initial benzonase treatment steps, a good representation of all the eight bacterial genera in the mock standard was shown. It is therefore imperative that researchers fully understand the importance of each step in any DNA extraction protocol and its impact on the bacterial community of their samples.

We showed no impact of DNA extraction kit on alpha diversity (reported using the Shannon diversity index), as has previously been shown on salivary bacterial communities [14]. When considering beta diversity (reported using Bray–Curtis dissimilarity index), samples clustered together based on donor, irrespective of the DNA extraction kit used or whether HM was skimmed or not prior to DNA extraction. A study evaluating variation in human gut microbiota profiles due to DNA extraction methods confirmed this. In this study, the main source of variation in a dissimilarity matrix was related to donor, followed by the DNA extraction method [19]. 

Another study by Wesolowska-Andersen et al. (2014), which assessed the effect of bacterial DNA extraction method from feces, observed a higher inter-individual variation than that seen for the extraction method [63]. A recent report that evaluated the effect of DNA extraction methodology on HM samples also found that inter-sample differences were a greater contributor to variation in HM bacterial composition than the DNA extraction method [18].

We observed in our study that DNA extraction kits had an impact, albeit relatively minor, on the relative abundances of specific bacterial taxa from extracted DNA of samples. Other studies have also observed significant differences in relative abundances of bacterial taxa based on DNA extraction methods for fecal specimens [63,64].

Though a major strength of this study was the use of mock microbial community, this study had a number of limitations. One is that the bacterial composition in the commercial mock community is not a true reflection of microbes usually found in HM. However, seven bacterial genera that are commonly isolated in HM were represented in the mock standard at the genus level. A further limitation is associated with the use of 16S rRNA gene-targeted sequencing, which gives low phylogenetic resolution power of bacteria up to the species level. Additionally, the mock microbial community was not extracted independently and processed by itself. Lastly, the “theoretical” bacterial profiles may differ from our experimental profiles due to factors such as the use of different 16S gene variable region and sequencing approaches.

## 5. Conclusions

In summary, we have shown that DNA extraction method, but not lipids removal, has an important influence on characterization of the HM bacteriome by 16S rRNA sequencing. Detailed understanding of the impact of individual steps within an extraction procedure is required to prevent significant bias in determining the composition of the HM bacteriome. 

## Supplementary Materials

The following are available online at https://www.mdpi.com/2409-9279/3/2/39/s1, Figure S1: DNA yield and purity based on skim milk and whole milk, Figure S2: 16S qPCR DNA concentration based on kit and milk type (skim milk and whole milk), Figure S3: Complete linkage hierarchical clustering of human milk spiked with the microbial mock community standard, Figure S4: 16S gene copy number/uL values of spiked and un-spiked human milk samples, Figure S5: Alpha diversity rarefaction curve, Figure S6: Relative abundances of bacterial composition in un-spiked human milk samples at genus level, Figure S7: Log ratio biplot human of human milk bacterial abundances at the phylum level, Figure S8: Log ratio biplot human of human milk bacterial abundances at the genus level, Table S1: Zymobiomics™ microbial community standard composition as per manufacturer’s specifications, Table S2: Primers, probes and cycling conditions, Table S3: Bacterial 16S DNA concentration in the spiked and un-spiked human milk sample, Table S4: Beta diversity (Bray–Curtis dissimilarity index) measuring dissimilarity between Zymobiomics microbial community standard (ZMCS) and the milk type, for each of the kits, Table S5: Differential abundances of statistically significant bacterial taxa in relation to extraction kits and milk type in un-spiked milk samples, Table S6: Reproducibility of extractions tested using multiple R-squared.

## References

[1] Asnicar F., Manara S., Zolfo M., Truong D.T., Scholz M., Armanini F., Ferretti P., Gorfer V., Pedrotti A., Tett A. (2017). Studying vertical microbiome transmission from mothers to infants by strain-level metagenomic profiling. mSystems.

[2] Civardi E., Garofoli F., Tzialla C., Paolillo P., Bollani L., Stronati M. (2013). Microorganisms in human milk: Lights and shadows. J. Matern. Fetal Neonatal Med..

[3] Jimenéz E., Fernández L., Maldonado A., Martin R., Olivares M., Xaus J., Rodriguez J.M. (2008). Oral administration of lactobacillus strains isolated from breast milk as an alternative for the treatment of infectious mastitis during lactation. Appl. Environ. Microbiol..

[4] Fernández L., Cárdenas N., Arroyo R., Manzano S., Jiménez E., Martín V., Rodríguez J.M. (2016). Prevention of infectious mastitis by oral administration of lactobacillus salivarius ps2 during late pregnancy. Clin. Infect. Dis..

[5] Yuan S., Cohen D.B., Ravel J., Abdo Z., Forney L.J. (2012). Evaluation of methods for the extraction and purification of DNA from the human microbiome. PLoS ONE.

[6] Fouhy F., Ross R.P., Fitzgerald G., Stanton C., Cotter P.D. (2012). Composition of the early intestinal microbiota:Knowledge, knowledge gaps and the use of high-throughput sequencing to address these gaps. Gut Microbes.

[7] Ojo-Okunola A., Nicol M., du Toit E. (2018). Human breast milk bacteriome in health and disease. Nutrients.

[8] Claesson M.J., Wang Q., O’sullivan O., Greene-Diniz R., Cole J.R., Ross R.P., O’Toole P.W. (2010). Comparison of two next-generation sequencing technologies for resolving highly complex microbiota composition using tandem variable 16s rrna gene regions. Nucleic Acids Res..

[9] Salipante S.J., Sengupta D.J., Rosenthal C., Costa G., Spangler J., Sims E.H., Jacobs M.A., Miller S.I., Hoogestraat D.R., Cookson B.T. (2013). Rapid 16s rrna next-generation sequencing of polymicrobial clinical samples for diagnosis of complex bacterial infections. PLoS ONE.

[10] Ariefdjohan M.W., Savaiano D.A., Nakatsu C.H. (2010). Comparison of DNA extraction kits for pcr-dgge analysis of human intestinal microbial communities from fecal specimens. Nutr. J..

[11] Claassen S., du Toit E., Kaba M., Moodley C., Zar H.J., Nicol M.P. (2013). A comparison of the efficiency of five different commercial DNA extraction kits for extraction of DNA from faecal samples. J. Microbiol. Methods.

[12] Chen H., Rangasamy M., Tan S.Y., Wang H., Siegfried B.D. (2010). Evaluation of five methods for total DNA extraction from western corn rootworm beetles. PLoS ONE.

[13] Miller D.N., Bryant J.E., Madsen E.L., Ghiorse W.C. (1999). Evaluation and optimization of DNA extraction and purification procedures for soil and sediment samples. Appl. Environ. Microbiol..

[14] Lazarevic V., Gaia N., Girard M., Francois P., Schrenzel J. (2013). Comparison of DNA extraction methods in analysis of salivary bacterial communities. PLoS ONE.

[15] Ó Cuív P., Aguirre de Cárcer D., Jones M., Klaassens E.S., Worthley D.L., Whitehall V.L.J., Kang S., McSweeney C.S., Leggett B.A., Morrison M. (2011). The effects from DNA extraction methods on the evaluation of microbial diversity associated with human colonic tissue. Microb. Ecol..

[16] Gill C., van de Wijgert J.H., Blow F., Darby A.C. (2016). Evaluation of lysis methods for the extraction of bacterial DNA for analysis of the vaginal microbiota. PLoS ONE.

[17] Usman T., Yu Y., Liu C., Fan Z., Wang Y. (2014). Comparison of methods for high quantity and quality genomic DNA extraction from raw cow milk. Genet. Mol. Res..

[18] Douglas C.A., Ivey K.L., Papanicolas L.E., Best K.P., Muhlhausler B.S., Rogers G.B. (2020). DNA extraction approaches substantially influence the assessment of the human breast milk microbiome. Sci. Rep..

[19] Wagner Mackenzie B., Waite D.W., Taylor M.W. (2015). Evaluating variation in human gut microbiota profiles due to DNA extraction method and inter-subject differences. Front. Microbiol..

[20] Hunt K.M., Foster J.A., Forney L.J., Schutte U.M., Beck D.L., Abdo Z., Fox L.K., Williams J.E., McGuire M.K., McGuire M.A. (2011). Characterization of the diversity and temporal stability of bacterial communities in human milk. PLoS ONE.

[21] Urbaniak C., Angelini M., Gloor G.B., Reid G. (2016). Human milk microbiota profiles in relation to birthing method, gestation and infant gender. Microbiome.

[22] Cabrera-Rubio R., Collado M.C., Laitinen K., Salminen S., Isolauri E., Mira A. (2012). The human milk microbiome changes over lactation and is shaped by maternal weight and mode of delivery. Am. J. Clin. Nutr..

[23] Macherey-Nagel Genomic DNA from Lipid-Rich Tissue: User Manual [Internet]. https://www.mn-net.com/Portals/8/attachments/Redakteure_Bio/Protocols/Genomic%20DNA/UM_gDNALipidTissue.pdf.

[24] Lucey J.A., Tamehana M., Singh H., Munro P.A. (1998). Effect of interactions between denatured whey proteins and casein micelles on the formation and rheological properties of acid skim milk gels. J. Dairy Res..

[25] Bogaert D., Keijser B., Huse S., Rossen J., Veenhoven R., van Gils E., Bruin J., Montijn R., Bonten M., Sanders E. (2011). Variability and diversity of nasopharyngeal microbiota in children: A metagenomic analysis. PLoS ONE.

[26] Wu L., Wen C., Qin Y., Yin H., Tu Q., Van Nostrand J.D., Yuan T., Yuan M., Deng Y., Zhou J. (2015). Phasing amplicon sequencing on illumina miseq for robust environmental microbial community analysis. BMC Microbiol..

[27] Claassen-Weitz S., Gardner-Lubbe S., Nicol P., Botha G., Mounaud S., Shankar J., Nierman W.C., Mulder N., Budree S., Zar H.J. (2018). Hiv-exposure, early life feeding practices and delivery mode impacts on faecal bacterial profiles in a south african birth cohort. Sci. Rep..

[28] Ojo-Okunola A., Claassen-Weitz S., Mwaikono K.S., Gardner-Lubbe S., Stein D.J., Zar H.J., Nicol M.P., du Toit E. (2019). Influence of socio-economic and psychosocial profiles on the human breast milk bacteriome of south african women. Nutrients.

[29] Caporaso J.G., Lauber C.L., Walters W.A., Berg-Lyons D., Lozupone C.A., Turnbaugh P.J., Fierer N., Knight R. (2011). Global patterns of 16s rrna diversity at a depth of millions of sequences per sample. Proc. Natl. Acad. Sci. USA.

[30] Illumina P. (2014). Miseq^®^ System User Guide.

[31] Andrews S. (2010). Fastqc: A Quality Control Tool for High Throughput Sequence Data.

[32] Edgar R.C. (2013). Uparse: Highly accurate otu sequences from microbial amplicon reads. Nat. Methods.

[33] Edgar R.C. (2010). Search and clustering orders of magnitude faster than blast. Bioinformatics.

[34] Wang Q., Garrity G.M., Tiedje J.M., Cole J.R. (2007). Naive bayesian classifier for rapid assignment of rrna sequences into the new bacterial taxonomy. Appl. Environ. Microbiol..

[35] Caporaso J.G., Kuczynski J., Stombaugh J., Bittinger K., Bushman F.D., Costello E.K., Fierer N., Pena A.G., Goodrich J.K., Gordon J.I. (2010). Qiime allows analysis of high-throughput community sequencing data. Nat. Methods.

[36] Quast C., Pruesse E., Yilmaz P., Gerken J., Schweer T., Yarza P., Peplies J., Glockner F.O. (2013). The silva ribosomal rna gene database project: Improved data processing and web-based tools. Nucleic Acids Res..

[37] Team R.C. (2017). R: A Language and Environment for Statistical Computing.

[38] Hartigan J.A. (1975). Clustering Algorithms.

[39] Murtagh F. (1985). Multidimensional clustering algorithms. Compstat Lectures.

[40] Bray J.R., Curtis J.T. (1957). An ordination of the upland forest communities of southern wisconsin. Ecol. Monogr..

[41] Oksanen J., Blanchet F., Kindt R., Legendre P., Minchin P., O’Hara R., Simpson G., Solymos P., Stevens M., Wagner H. Vegan: Community Ecology Package. R Package Version 2.4-4.

[42] Hill M.O. (1973). Diversity and evenness: A unifying notation and its consequences. Ecology.

[43] Chambers J.M., Hastie T.J. (1992). Statistical Models in s.

[44] Greenacre M.J. (2010). Biplots in Practice.

[45] Gower J.C., Lubbe S.G., Le Roux N.J. (2011). Understanding Biplots.

[46] Fernandes A.D., Reid J.N., Macklaim J.M., McMurrough T.A., Edgell D.R., Gloor G.B. (2014). Unifying the analysis of high-throughput sequencing datasets: Characterizing rna-seq, 16s rrna gene sequencing and selective growth experiments by compositional data analysis. Microbiome.

[47] Devroye L. (1986). Sample-based non-uniform random variate generation. Proceedings of the 18th Conference on Winter Simulation.

[48] McCullagh P., Nelder J.A. (1989). Generalized Linear Models.

[49] Benjamini Y., Hochberg Y. (1995). Controlling the false discovery rate: A practical and powerful approach to multiple testing. J. R. Stat. Soc. Ser. B (Methodol.).

[50] Yandell B.S. (1997). Practical Data Analysis for Designed Experiments.

[51] Chambers J.M., Cleveland W.S., Kleiner B., Tukey P.A. (1983). Graphical Methods for Data Analysis.

[52] Jost T., Lacroix C., Braegger C., Chassard C. (2013). Assessment of bacterial diversity in breast milk using culture-dependent and culture-independent approaches. Br. J. Nutr..

[53] Mathay C., Hamot G., Henry E., Georges L., Bellora C., Lebrun L., de Witt B., Ammerlaan W., Buschart A., Wilmes P. (2015). Method optimization for fecal sample collection and fecal DNA extraction. Biopreserv. Biobank..

[54] Abusleme L., Hong B.Y., Dupuy A.K., Strausbaugh L.D., Diaz P.I. (2014). Influence of DNA extraction on oral microbial profiles obtained via 16s rrna gene sequencing. J. Oral Microbiol..

[55] Terrat S., Christen R., Dequiedt S., Lelievre M., Nowak V., Regnier T., Bachar D., Plassart P., Wincker P., Jolivet C. (2012). Molecular biomass and metataxogenomic assessment of soil microbial communities as influenced by soil DNA extraction procedure. Microb. Biotechnol..

[56] Cremonesi P., Castiglioni B., Malferrari G., Biunno I., Vimercati C., Moroni P., Morandi S., Luzzana M. (2006). Technical note: Improved method for rapid DNA extraction of mastitis pathogens directly from milk. J. Dairy Sci..

[57] Jervis-Bardy J., Leong L.E.X., Marri S., Smith R.J., Choo J.M., Smith-Vaughan H.C., Nosworthy E., Morris P.S., O’Leary S., Rogers G.B. (2015). Deriving accurate microbiota profiles from human samples with low bacterial content through post-sequencing processing of illumina miseq data. Microbiome.

[58] Glassing A., Dowd S.E., Galandiuk S., Davis B., Chiodini R.J. (2016). Inherent bacterial DNA contamination of extraction and sequencing reagents may affect interpretation of microbiota in low bacterial biomass samples. Gut Pathog..

[59] Salter S.J., Cox M.J., Turek E.M., Calus S.T., Cookson W.O., Moffatt M.F., Turner P., Parkhill J., Loman N.J., Walker A.W. (2014). Reagent and laboratory contamination can critically impact sequence-based microbiome analyses. BMC Biol..

[60] Willner D., Daly J., Whiley D., Grimwood K., Wainwright C.E., Hugenholtz P. (2012). Comparison of DNA extraction methods for microbial community profiling with an application to pediatric bronchoalveolar lavage samples. PLoS ONE.

[61] Wen Y., Xiao F., Wang C., Wang Z. (2016). The impact of different methods of DNA extraction on microbial community measures of balf samples based on metagenomic data. Am. J. Transl. Res..

[62] Nelson M.T., Pope C.E., Marsh R.L., Wolter D.J., Weiss E.J., Hager K.R., Vo A.T., Brittnacher M.J., Radey M.C., Hayden H.S. (2019). Human and extracellular DNA depletion for metagenomic analysis of complex clinical infection samples yields optimized viable microbiome profiles. Cell Rep..

[63] Wesolowska-Andersen A., Bahl M.I., Carvalho V., Kristiansen K., Sicheritz-Pontén T., Gupta R., Licht T.R. (2014). Choice of bacterial DNA extraction method from fecal material influences community structure as evaluated by metagenomic analysis. Microbiome.

[64] Kennedy N.A., Walker A.W., Berry S.H., Duncan S.H., Farquarson F.M., Louis P., Thomson J.M. (2014). The impact of different DNA extraction kits and laboratories upon the assessment of human gut microbiota composition by 16s rrna gene sequencing. PLoS ONE.

