# Supplementary material for: The Influence of DNA Extraction and Lipid Removal on Human Milk Bacterial Profiles
Source: Methods Protoc. 2020 May 15;3(2):39. doi: 10.3390/mps3020039 (PMC7359716; doi:10.3390/mps3020039)
Supplement: Supplementary file 1 [file mps-03-00039-s001.zip › Figure S2.pdf]

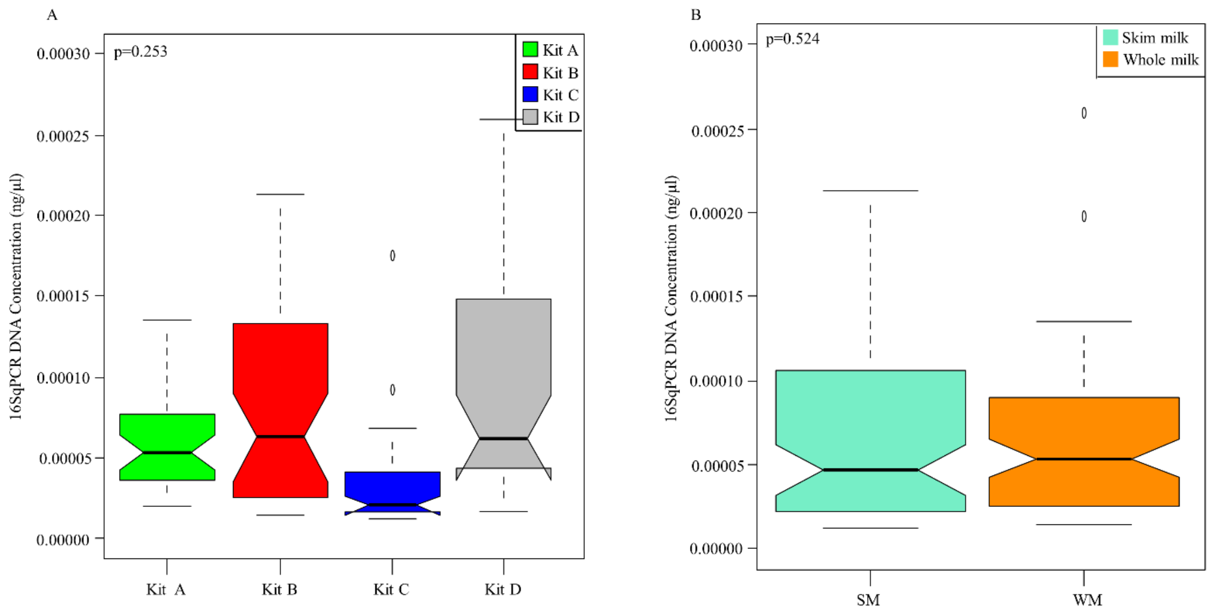

S2 Figure: 16SqPCR DNA concentration based on kit and milk type (skim milk and whole milk). Notched box plots showing the 16S qPCR DNA concentration obtained by using (A) the kits, and (B) skim and whole milk. The notched box signifies the 75% (upper) and 25% (lower) quartile showing the distribution of 50% of the samples. The line inside the box plot represents the median, and the notch the 95% confidence interval for the median. The whiskers (top and bottom) represent the maximum and minimum values. Outliers, which are beyond 1.5 times the interquartile range above the maximum value and below the minimum value, are shown with open circles. SM=skim milk, WM=whole milk.
