# Supplementary material for: The Influence of DNA Extraction and Lipid Removal on Human Milk Bacterial Profiles
Source: Methods Protoc. 2020 May 15;3(2):39. doi: 10.3390/mps3020039 (PMC7359716; doi:10.3390/mps3020039)
Supplement: Supplementary file 1 [file mps-03-00039-s001.zip › Figure S3.pdf]

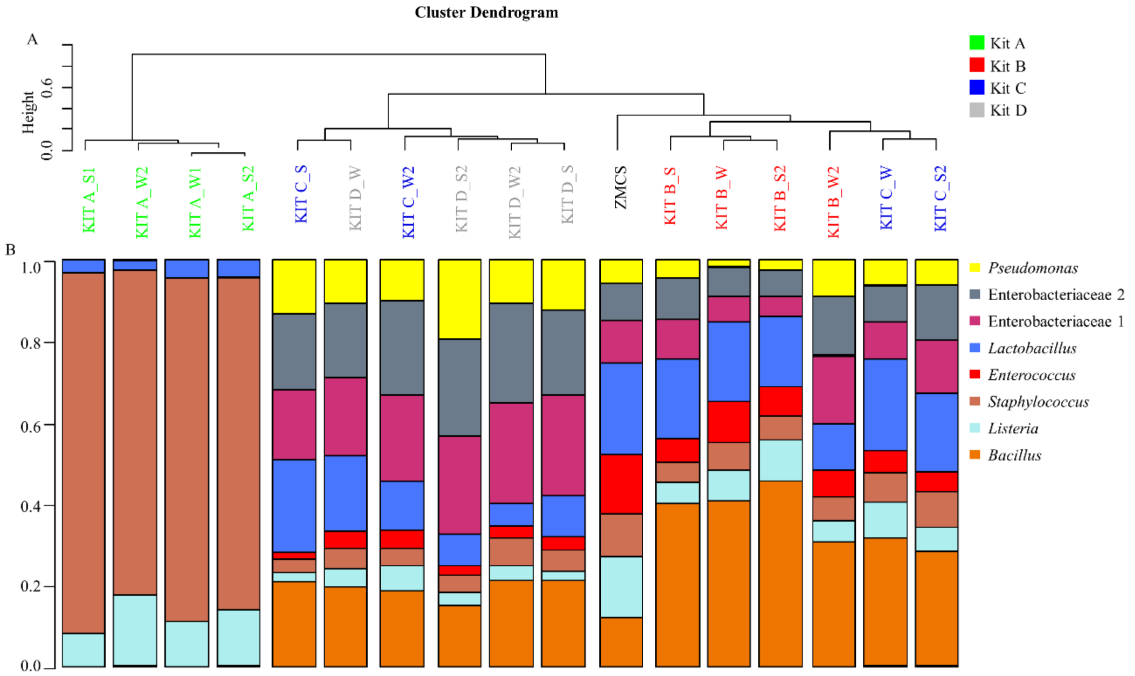


**S3 Figure: Complete linkage hierarchical clustering of human breast milk spiked with the microbial mock community standard.** (A) Dendogram of bacterial profiles present in the HBM sample spiked with ZMCS processed as skim and whole milk using four DNA extraction kits. (B) Relative abundances of ZMCS bacterial composition in the HBM sample spiked with ZMCS. Each column represents milk type (skim or whole) extracted by one of the four kits. W: whole milk, S: skim milk; S2 and W2: replicates of S and W respectively. ZMCS: Zymobiomics Microbial Community Standard reference ("theoretical" bacterial profile) specified by the manufacturer.
