# Supplementary material for: The Influence of DNA Extraction and Lipid Removal on Human Milk Bacterial Profiles
Source: Methods Protoc. 2020 May 15;3(2):39. doi: 10.3390/mps3020039 (PMC7359716; doi:10.3390/mps3020039)
Supplement: Supplementary file 1 [file mps-03-00039-s001.zip › Figure S4.pdf]

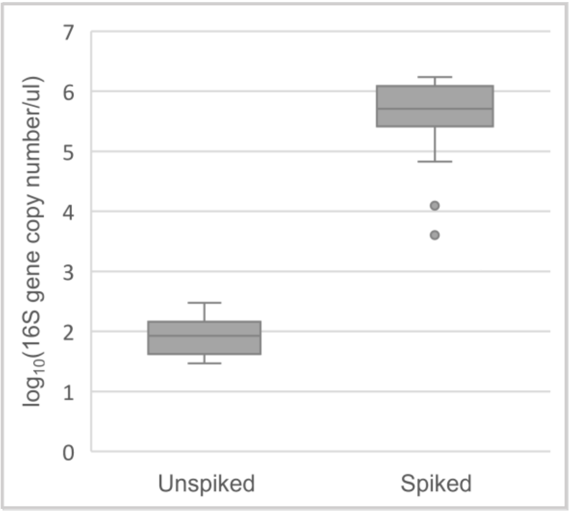

S4 Figure: 16S gene copy number/ul values of spiked and un-spiked human breast milk samples. The box signifies the 75% (upper) and 25% (lower) quartile showing the distribution of 50% of the samples. The line inside the box plot represents the median, and the whiskers (top and bottom) represent the maximum and minimum values. Outliers, which are beyond 1.5 times the interquartile range above the maximum value and below the minimum value, are shown with open circles.
