# Supplementary material for: The Influence of DNA Extraction and Lipid Removal on Human Milk Bacterial Profiles
Source: Methods Protoc. 2020 May 15;3(2):39. doi: 10.3390/mps3020039 (PMC7359716; doi:10.3390/mps3020039)
Supplement: Supplementary file 1 [file mps-03-00039-s001.zip › Figure S5.pdf]

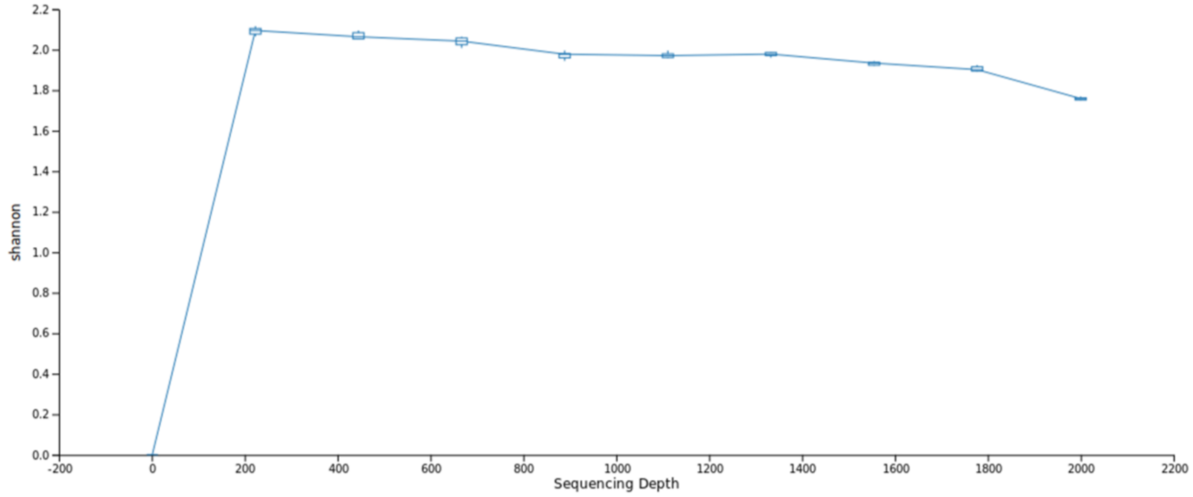

Figure S5: Alpha diversity rarefaction curve. A line graph relating the sequencing depth to Shannon diversity index of sequenced un-spiked breast milk samples at a sub-sampling depth of 2100.
