# Supplementary material for: The Influence of DNA Extraction and Lipid Removal on Human Milk Bacterial Profiles
Source: Methods Protoc. 2020 May 15;3(2):39. doi: 10.3390/mps3020039 (PMC7359716; doi:10.3390/mps3020039)
Supplement: Supplementary file 1 [file mps-03-00039-s001.zip › Figure S6.pdf]

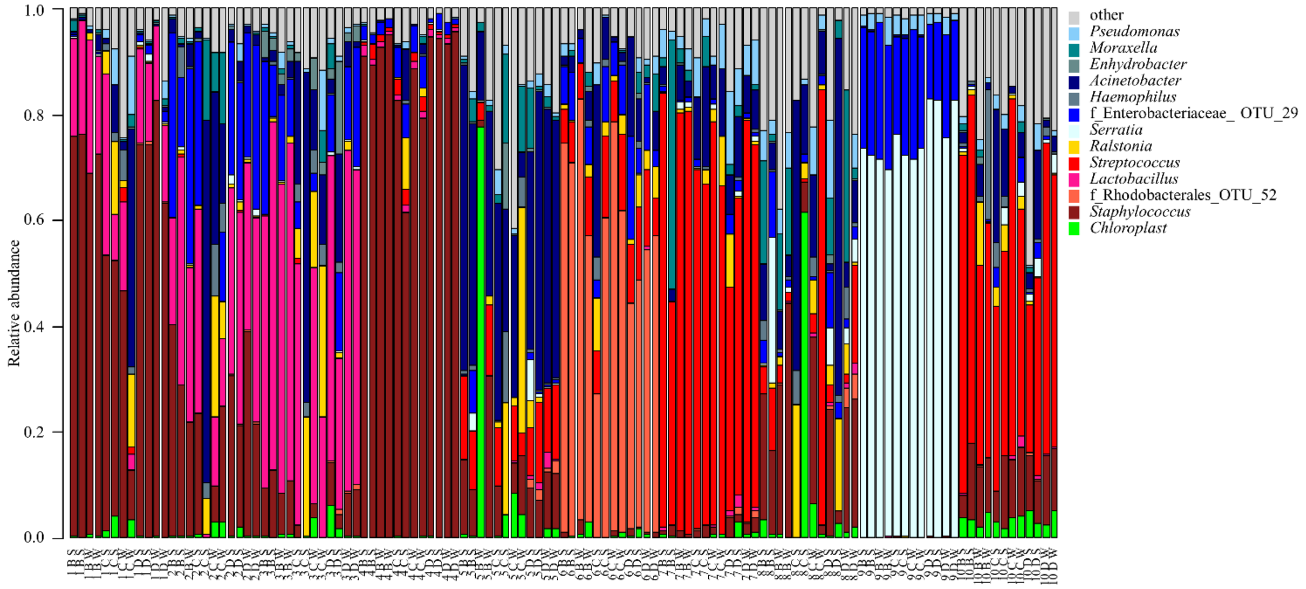


**S6 Figure**: **Relative abundances of bacterial composition in un-spiked human breast milk samples at genus level.** Each bar represents a sample and each coloured box, a bacterium. Genera less than 0.5% abundance in a given sample are included in the “other” at the top of the graph (grey boxes).
