# Supplementary material for: The Influence of DNA Extraction and Lipid Removal on Human Milk Bacterial Profiles
Source: Methods Protoc. 2020 May 15;3(2):39. doi: 10.3390/mps3020039 (PMC7359716; doi:10.3390/mps3020039)
Supplement: Supplementary file 1 [file mps-03-00039-s001.zip › Figure S7.pdf]

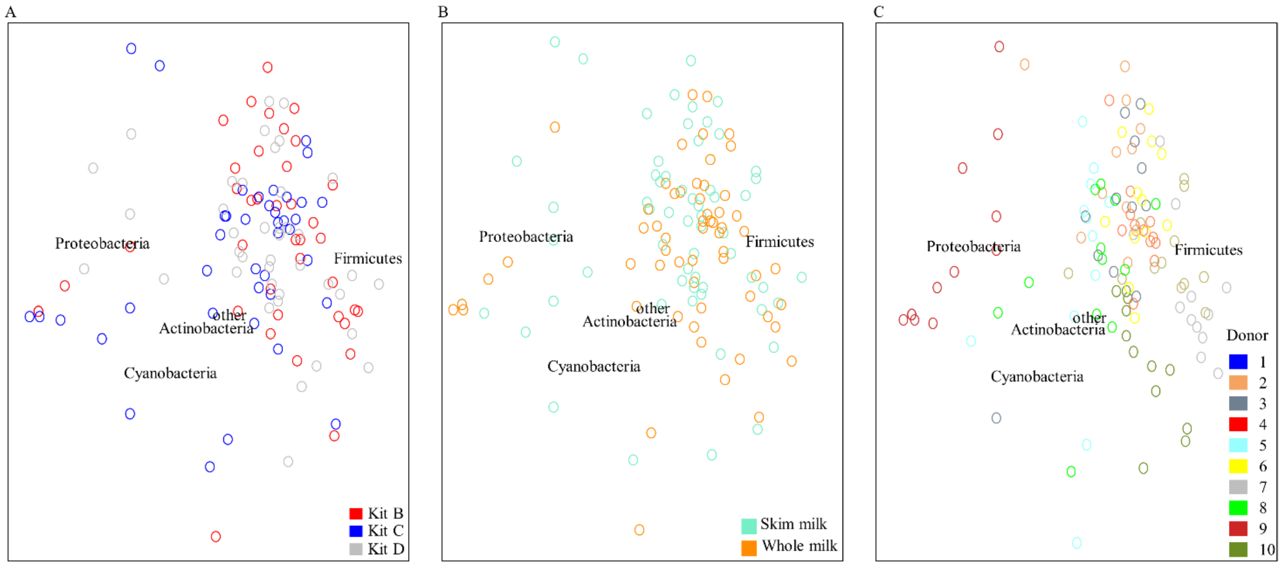

S7 Figure: Log ratio biplot human of human breast milk bacterial abundances at the phylum level. Samples are coloured according to (a) extraction kit used, and (b) whole and skim milk (c) donors. Points (circles) that cluster together are similar in bacterial composition and abundance.
