# Supplementary material for: The Influence of DNA Extraction and Lipid Removal on Human Milk Bacterial Profiles
Source: Methods Protoc. 2020 May 15;3(2):39. doi: 10.3390/mps3020039 (PMC7359716; doi:10.3390/mps3020039)
Supplement: Supplementary file 1 [file mps-03-00039-s001.zip › Table S1.pdf]

S1 Table: Zymobiomics™ microbial community standard composition as per manufacturer's specifications.

Bacterial taxa	gDNA abundance (%)	mOTU abundance (%)	Gram stain
Pseudomonas spp.	12%	4.6	-
Enterobacteriaceae 1	12%	11.3	-
Enterobacteriaceae 2	12%	10.0	-
Listeria spp.	12%	15.9	+
Staphylococcus spp.	12%	13.3	+
Lactobacillus spp.	12%	18.8	+
Enterococcus spp.	12%	10.4	+
Bacillus spp.	12%	15.7	+
