# Supplementary material for: The Influence of DNA Extraction and Lipid Removal on Human Milk Bacterial Profiles
Source: Methods Protoc. 2020 May 15;3(2):39. doi: 10.3390/mps3020039 (PMC7359716; doi:10.3390/mps3020039)
Supplement: Supplementary file 1 [file mps-03-00039-s001.zip › Table S2.pdf]

S2 Table: Primers, probes and cycling conditions

		Forward primer	Reverse primer	Probe	PCR cycling conditions
**A**	qPCR	16S-F1(5′-CGA AAG CGT GGG GAG CAA A -3′)	16S-R1(5′-GTT CGT ACT CCC CAG GCG G-3′)	16S-P1 (FAM- ATT AGA TAC CCT GGT AGT CCA –MGB	45 cycles<br>50 ºC 95 ºC 95 ºC 60 °C 72 °C<br>2min 10min 15s 60s 1s
**B**	Short PCR	(515Fshort) GTGCCAGCHGCYGCGGT	(806Rshort) GGACTACNNGGGTNTCTAAT	-	10 cycles<br>95 ºC 95 ºC 50 °C 72 °C 72 °C 4 °C<br>3min 30s 30s 1s 5min ∞
**C**	Long PCR	(515Flong) GTGCCAGCHGCYGCGGT	(806Rlong_barcoded) GGACTACNNGGGTNTCTAAT	-	30 cycles<br>95 ºC 95 ºC 50 °C 72 °C 72 °C 4 °C<br>3min 30s 30s 1s 5min ∞
