# Supplementary material for: The Influence of DNA Extraction and Lipid Removal on Human Milk Bacterial Profiles
Source: Methods Protoc. 2020 May 15;3(2):39. doi: 10.3390/mps3020039 (PMC7359716; doi:10.3390/mps3020039)
Supplement: Supplementary file 1 [file mps-03-00039-s001.zip › Table S3.pdf]

S3 Table. Bacterial 16S DNA concentration in the spiked and un-spiked human breast milk sample.

Sample_ID	Spike/unspike_Kit	Donor sample	WM/SM	Template DNA 16SqPCR concentration (ng/ul)	Median 16S qPCR DNA concentration	%16S DNA in HBM sample	16S gene copy number/ul
BM_AO_KITdnam_extractioncontrolspike_1_kitcomparison_P3_A06	Spiked_Kit A	1	WM	0.251124139	0.195487886	99.975%	354043.0421
BM_AO_KITdnam_extractioncontrolspike_1_kitcomparison_P3_A07	Spiked_Kit A	1	WM	0.175701367			247709.5464
BM_AO_KITdnam_extractioncontrolspike_1_kitcomparison_P3_B06	Spiked_Kit A	1	SM	0.215274404			303500.9107
BM_AO_KITdnam_extractioncontrolspike_1_kitcomparison_P3_B07	Spiked_Kit A	1	SM	0.047838204			67443.86864
BM_AO_KITdnam_skim_1_kitcomparison_P3_C01	Unspiked_Kit A	1	SM	0.000047258	0.000047105	0.024%	66.62577892
BM_AO_KITdnam_skim_1_kitcomparison_P3_C08	Unspiked_Kit A	1	SM	0.000046952			66.19495263
BM_AO_KITdnam_whole_1_kitcomparison_P3_A01	Unspiked_Kit A	1	WM	0.000068726			96.89241007
BM_AO_KITdnam_whole_1_kitcomparison_P3_A08	Unspiked_Kit A	1	WM	0.000045226			63.76078519
BM_AO_KITqias_extractioncontrol_1_kitcomparison_P4_A06	Spiked_Kit C	1	WM	0.332685221	0.151318187	99.983%	469030.529
BM_AO_KITqias_extractioncontrol_1_kitcomparison_P4_A07	Spiked_Kit C	1	WM	0.293789111			414193.5186
BM_AO_KITqias_extractioncontrol_1_kitcomparison_P4_B06	Spiked_Kit C	1	SM	0.002849745			4017.664268
BM_AO_KITqias_extractioncontrol_1_kitcomparison_P4_B07	Spiked_Kit C	1	SM	0.008847263			12473.16112
BM_AO_KITqias_skim_1_kitcomparison_P4_C01	Unspiked_Kit C	1	SM	0.000060109	0.000025238	0.016%	84.7437381
BM_AO_KITqias_skim_1_kitcomparison_P4_C08	Unspiked_Kit C	1	SM	0.000024687			34.80428223
BM_AO_KITqias_whole_1_kitcomparison_P4_A01	Unspiked_Kit C	1	WM	0.000025790			36.35925842
BM_AO_KITqias_whole_1_kitcomparison_P4_A08	Unspiked_Kit C	1	WM	0.000020727			29.22103109
BM_AO_KITzymob_extractioncontrol_1_kitcomparison_P4_G06	Spiked_Kit D	1	WM	1.214408365	0.843423774	99.989%	1712112.716
BM_AO_KITzymob_extractioncontrol_1_kitcomparison_P4_G07	Spiked_Kit D	1	WM	0.397619204			560576.5863
BM_AO_KITzymob_extractioncontrol_1_kitcomparison_P4_H06	Spiked_Kit D	1	SM	0.792653652			1117509.098
BM_AO_KITzymob_extractioncontrol_1_kitcomparison_P4_H07	Spiked_Kit D	1	SM	0.894193897			1260663.863
BM_AO_KITzymob_skim_1_kitcomparison_P4_G01	Unspiked_Kit D	1	SM	0.000066957	0.000087592	0.01%	94.39819402
BM_AO_KITzymob_skim_1_kitcomparison_P4_G08	Unspiked_Kit D	1	SM	0.000108227			152.58203
BM_AO_KITzymob_whole_1_kitcomparison_P4_E01	Unspiked_Kit D	1	WM	0.000059373			83.70631017
BM_AO_KITzymob_whole_1_kitcomparison_P4_E08	Unspiked_Kit D	1	WM	0.000198393			279.700552
BM_AO_KITzymo_extractioncontrol_1_kitcomparison_P3_G06	Spiked_Kit B	1	WM	0.703367972	0.898759654	99.984%	991631.2204
BM_AO_KITzymo_extractioncontrol_1_kitcomparison_P3_G07	Spiked_Kit B	1	WM	1.094151336			1542570.415
BM_AO_KITzymo_extractioncontrol_1_kitcomparison_P3_H06	Spiked_Kit B	1	SM	0.654916126			923322.2206
BM_AO_KITzymo_extractioncontrol_1_kitcomparison_P3_H07	Spiked_Kit B	1	SM	1.123935714			1584561.408
BM_AO_KITzymo_skim_1_kitcomparison_P3_G01	Unspiked_Kit B	1	SM	0.000194474	0.000141692	0.015%	274.1758909
BM_AO_KITzymo_skim_1_kitcomparison_P3_G08	Unspiked_Kit B	1	SM	0.000211923			298.7761734
BM_AO_KITzymo_whole_1_kitcomparison_P3_E01	Unspiked_Kit B	1	WM	0.000025031			35.28893217
BM_AO_KITzymo_whole_1_kitcomparison_P3_E08	Unspiked_Kit B	1	WM	0.000088910			125.3482042

ZMCS: Zymobiomics Microbial Community Standard; HBM: Human breast milk; SM: Skim milk, WM: Whole milk
