# Supplementary material for: The Influence of DNA Extraction and Lipid Removal on Human Milk Bacterial Profiles
Source: Methods Protoc. 2020 May 15;3(2):39. doi: 10.3390/mps3020039 (PMC7359716; doi:10.3390/mps3020039)
Supplement: Supplementary file 1 [file mps-03-00039-s001.zip › Table S4.pdf]

S4 Table: Beta diversity (Bray-Curtis dissimilarity index) measuring dissimilarity between Zymobiomics microbial community standard (ZMCS) and the milk type, for each of the kits.

	**Milk type**	
Kits	Skim milk	Whole milk
Kit A vs. ZMCS	0.29	0.25
Kit B vs. ZMCS	0.06	0.16
Kit C vs. ZMCS	0.16	0.16
Kit D vs. ZMCS	0.11	0.20
