# Supplementary material for: The Influence of DNA Extraction and Lipid Removal on Human Milk Bacterial Profiles
Source: Methods Protoc. 2020 May 15;3(2):39. doi: 10.3390/mps3020039 (PMC7359716; doi:10.3390/mps3020039)
Supplement: Supplementary file 1 [file mps-03-00039-s001.zip › Table S5.pdf]

S5 Table: Differential abundances of statistically significant bacteria taxa in relation to extraction kits and milk type in un-spiked breast milk samples.

Taxonomy level	Kit B	Kit C	Kit D	p-value
KITS				
Proteobacteria	0.3436	0.4761	0.3772	0.005
Alphaproteobacteria	0.0135	0.0162	0.0093	0.001
Sphingomonadales	0.0058	0.0087	0.0039	<0.001
Rhodobacterales				
OTU_52	0.0712	0.0564	0.0561	0.035
Betaproteobacteria	0.0294	0.089	0.0271	<0.001
Burkholderiales	0.0244	0.0856	0.0242	<0.001
Burkholderiaceae	0.007	0.0758	0.0176	<0.001
Ralstonia	0.007	0.075	0.0175	<0.001
Gammaproteobacteria				
Pseudomonadales	0.1157	0.2086	0.1607	<0.001
Pseudomonadaceae	0.0167	0.0259	0.024	0.016
Pseudomonas	0.0165	0.0259	0.024	0.016
Pasteurellales	0.1109	0.2205	0.0045	<0.001
Pasteurellaceae	0.011	0.2205	0.0045	<0.001
Haemophilus	0.011	0.022	0.0045	<0.001
Moraxellaceae				
Acinetobacter	0.0606	0.1544	0.1026	<0.001
Firmicutes				
Bacillus				
Lactobacillales				
Lactobacillaceae	0.1084	0.0632	0.1088	<0.001
Lactobacillus	0.1084	0.0632	0.1088	<0.001
WM/SM				
	Skim milk	Whole milk		
Pseudomonadales	0.1948	0.1288	--	0.009
Moraxellaceae	0.1721	0.1068	--	0.004
Enhydrobacter	0.0078	0.0039	--	0.002
Acinetobacter	0.1353	0.0767	--	0.003

Mean values of relative abundance proportions for statistically significant bacteria at different taxonomy levels are shown. *p*-values are Benjamini-Hochberg corrected. *p*-values are represented as '***' 0.001; '**' 0.01; and '*' 0.05. WM and SM represents whole milk and skim milk respectively.
