# Supplementary material for: The Influence of DNA Extraction and Lipid Removal on Human Milk Bacterial Profiles
Source: Methods Protoc. 2020 May 15;3(2):39. doi: 10.3390/mps3020039 (PMC7359716; doi:10.3390/mps3020039)
Supplement: Supplementary file 1 [file mps-03-00039-s001.zip › Table S6.pdf]

S6 Table: Reproducibility of extractions tested using multiple R-squared

	Multiple R-square	
	Whole milk	Skim milk
Kit B	0.4908	0.7581
Kit C	0.7421	0.4751
Kit D	0.9483	0.7976
